# ASL mRNA-LNP Therapeutic for the Treatment of Argininosuccinic Aciduria Enables Survival Benefit in a Mouse Model

**DOI:** 10.3390/biomedicines11061735

**Published:** 2023-06-16

**Authors:** Owen Daly, Azita Josefine Mahiny, Sara Majeski, Kevin McClintock, Julia Reichert, Gábor Boros, Gábor Tamás Szabó, Jonas Reinholz, Petra Schreiner, Steve Reid, Kieu Lam, Marlen Lepper, Melanie Adler, Tracy Meffen, James Heyes, Katalin Karikó, Pete Lutwyche, Irena Vlatkovic

**Affiliations:** 1 Genevant Sciences Corporation, Vancouver, BC V5T 4T5, Canada; 2 BioNTech SE, An der Goldgrube 12, 55131 Mainz, Germany

**Keywords:** lipid nanoparticle-mRNA (LNP-mRNA), mRNA optimization, mRNA therapeutic, rare disease, argininosuccinic aciduria (ASA), argininosuccinate lyase deficiency (ASLD)

## Abstract

Argininosuccinic aciduria (ASA) is a metabolic disorder caused by a deficiency in argininosuccinate lyase (ASL), which cleaves argininosuccinic acid to arginine and fumarate in the urea cycle. ASL deficiency (ASLD) leads to hepatocyte dysfunction, hyperammonemia, encephalopathy, and respiratory alkalosis. Here we describe a novel therapeutic approach for treating ASA, based on nucleoside-modified messenger RNA (modRNA) formulated in lipid nanoparticles (LNP). To optimize ASL-encoding mRNA, we modified its cap, 5′ and 3′ untranslated regions, coding sequence, and the poly(A) tail. We tested multiple optimizations of the formulated mRNA in human cells and wild-type C57BL/6 mice. The ASL protein showed robust expression in vitro and in vivo and a favorable safety profile, with low cytokine and chemokine secretion even upon administration of increasing doses of ASL mRNA-LNP. In the ASL^Neo/Neo^ mouse model of ASLD, intravenous administration of the lead therapeutic candidate LNP-ASL CDS2 drastically improved the survival of the mice. When administered twice a week lower doses partially protected and 3 mg/kg LNP-ASL CDS2 fully protected the mice. These results demonstrate the considerable potential of LNP-formulated, modified ASL-encoding mRNA as an effective alternative to AAV-based approaches for the treatment of ASA.

## 1. Introduction

Argininosuccinic aciduria (ASA) is a rare autosomal recessive metabolic disorder affecting approximately 1 in 218,750 live births in the US [1]. ASL deficiency (ASLD) is caused by mutations in the ASL gene, located on human chromosome 7, locus 7q11.21 [2]. This enzyme (1) catalyzes the production of fumarate and arginine in the urea cycle, allowing degradation of excess nitrogen in the form of ammonia from the blood to urea that can be excreted from the organism, and (2) has a structural role in the citrulline–nitric oxide cycle, where together with other proteins forms part of a complex allowing nitric oxide (NO) and citrulline production from arginine by nitric oxide synthase (NOS) [3,4,5,6]. While the citrulline–nitric oxide cycle occurs normally in neurons, microglia, chondrocytes, macrophages, endothelial, epithelial, muscle cells, and hepatocytes [5,7], the urea cycle is restricted to periportal hepatocytes, the only cell type expressing all six necessary enzymes [8].

Mutated ASL enzyme leads to a loss of hepatocyte homeostasis and results in hyperammonemia, encephalopathy, and respiratory alkalosis [9]. While the liver is mainly affected, the kidneys and brain are also impacted by this disease [9,10]. In severe cases, the symptomatic onset of ASA typically occurs within the first two to three days of life, while a late-onset form of ASL deficiency can be triggered at almost any time by illness or stress. For example, a late-onset ASL deficiency that led to hyperammonemia was triggered by influenza infection in an adolescent patient [11]. The spectrum of disease severity is broad and depends on the residual activity of the ASL enzyme. While there is no cure for this medical condition, possible treatments include dietary protein restrictions to prevent endogenous protein catabolism, L-arginine hydrochloride or citrulline administration to maximize ammonia excretion, ammonia scavengers (e.g., sodium benzoate and sodium phenyl butylate/sodium phenylacetate), and liver transplantation [10,12]. Liver transplants as early as possible are considered the best option for ASL patients to prevent poor neurological outcomes and improve their quality of life; however, this is only possible for selected patients [13,14,15].

Liver-targeted adeno-associated viral (AAV)-based gene therapy was recently used as an approach for the treatment of ASL deficiency [16]. The authors of that study used an AAV8 vector expressing the human ASL gene under the control of the liver-specific thyroxine-binding globulin (TBG) promoter and evaluated therapeutic efficacy in ASA hypomorphic mice [16]. They found that neonatal administration extended the survival albeit not to wild-type levels, while administration to adolescent hypomorphic mice led to improved survival and weight gain at a high dose [16]. In a second AAV-based therapy study, the authors used an AAV8 vector carrying the murine ASL gene under the short version of the elongation factor 1 α (EFS) promoter [6]. They examined simultaneous brain and liver transfer in ASL hypomorphic mice and found improved survival and macroscopic features when adult mice were injected, while only a transient improvement was observed after neonatal injection. However, AAV gene therapy has limited durability in pediatric patients, can be associated with liver genotoxicity, and neutralizing antibodies can develop in response to AAV vectors making re-dosing less efficient [17,18,19,20].

Recently, two LNP-mRNA based vaccines were approved against coronavirus disease 2019 (COVID-19) [21,22]. The development of mRNA-based vaccines for COVID-19 led to its rapid advancement for other infectious diseases and cancer immunotherapies and the emergence of mRNA-based therapeutics coding for antibodies and RNA protein replacement therapies (reviewed in [23,24,25]). In this study, we evaluated the potential of mRNA therapeutics as a treatment for ASA. ASA is part of a group of inherited metabolic disorders in the urea cycle, which also includes Ornithine transcarbamylase deficiency (OTCD) and Citrullinemia. The underlying technology developed for ASA could have broader applications for other rare genetic disorders that are similar in nature. We engineered mRNA-based treatments consisting of an optimized ASL-modified mRNA, formulated in liver-targeted lipid nanoparticles (LNP). Utilizing both in vitro and in vivo models we administered and compared four optimized LNP-mRNAs and found robust ASL protein expression and low immunogenicity in human cells and in mice. Finally, we found improved survival and body weight in the ASL^Neo/Neo^ mouse model of ASLD after single and repetitive injections of our optimized ASL mRNA-LNP therapeutic.

## 2. Materials and Methods

### 2.1. Manufacturing of ASL mRNA-LNP

Manufacturing of the ASL mRNA (CDS1, CDS2 and CDS3) was performed by in vitro transcription using the MEGAscript T7 Transcription kit (Thermo Fisher Scientific, Waltham, MA, USA) in the presence of N1-methylpseudouridine (m1Ψ) triphosphate (TriLink, San Diego, CA, USA) instead of uridine-5′-triphosphate (UTP), from linearized DNA plasmid template encoding wild-type (CDS1) or codon-optimized (CDS2, CDS3) ASL harboring an α-globin 5’UTR and an AES and mtRNR1 3’UTR [26,27]. mRNA was capped co-transcriptionally using Cap1 ((m2 7,3′-O)Gppp(m2′-O)ApG; TriLink), while the poly(A) tail was encoded by the template. Prior to formulation, ASL mRNA was purified using cellulose chromatography [28]. Manufacturing of the ASL mRNA CDS4 was performed by in vitro transcription by TriLink, San Diego, CA, USA in the presence of pseudouridine-5′-triphosphate (ΨTP, TriLink) instead of uridine-5′-triphosphate (UTP), from linearized DNA plasmid template encoding codon-optimized ASL (CDS4), a minimal unstructured 5’UTR, and α-globin 3’UTR [29]. ASL mRNA CDS4 is enzymatically capped, and polyadenylated post-transcriptionally, then purified using HPLC by TriLink, San Diego, CA, USA. The mRNA concentration of all four tested ASL mRNA optimizations was measured on NanoDropTM 2000c spectrophotometer (Thermo Fisher Scientific, USA) and RNA integrity was determined using Bioanalyzer. The capping efficiency of ASL mRNA CDS1-3 was measured using a PAGE-based capping assay and for CDS4 using a TriLink Mass Spec-based assay. The amount of dsRNA contaminants was measured using a dot blot assay based on the J2 anti-dsRNA antibody (Scicons, Budapest, Hungary) as previously described in Baiersdörfer et al., 2018 [28] or for CDS4 using a slot blot assay (performed by TriLink, San Diego, CA, USA). In all cases, the quality of IVT mRNA passed the requirements. Cellulose-purified mRNA was encapsulated in LNPs using a controlled mixing process [30] in which an aqueous solution of mRNA at pH 5 was combined with an ethanolic solution of lipids, containing the PEG2000-C-DMA, an ionizable lipid, cholesterol, and 1,2-distearoyl-sn-glycero-3-phosphocholine (DSPC) at molar ratios of 1.6:54.6:32.8:10.9 [31,32]. Ethanol was removed by tangential flow ultrafiltration, followed by buffer exchange and concentration. The formulation was filtered through a 0.2 μm polyethersulfone membrane and aliquots were subsequently stored frozen at −80 °C in Tris-sucrose buffer, pH 8.0. Quality controls of LNP-ASL mRNA included measurements of particle size and polydispersity by dynamic light scattering (Malvern Nano ZS Zetasizer, Malvern, UK) and RNA encapsulation efficiency by the Quant-iT RiboGreen Assay (Life Technologies, Carlsbad, CA, USA). Lipoplex-unmodified RNA was manufactured as described in [33].

### 2.2. In Vitro Cell Culture and Transfections of LNP-mRNA

Chinese hamster ovary (CHO) and human embryonic kidney (HEK293) cells, (ATCC) were cultured under standard conditions in Ham’s F12 Medium (PAN Biotech, Aidenbach, Germany) and DMEM (Life Technologies) containing 10% non-heat inactivated fetal calf serum (FCS) at 37 °C, 5% CO_2_ and 37 °C, 7.5% CO_2_, respectively. The cells were plated at a density of 30,000 cells per well in a 96-well plate and transfected in quadruplicates with 0.1 µg/well of LNP-ASL mRNAs. Samples were collected at time points from 24 h up to 72 h after transfection by removal of supernatants and lysis of the cell layers in RIPA buffer containing protease inhibitors. Four wells per sample were pooled and centrifuged at 10,000 rpm for 12 min to remove cell debris. Samples were stored at −20 °C until further analysis.

Primary human hepatocytes (BioIVT, Westbury, NY, USA, were cultured in InVitroGRO CP and InVitroGRO HI media and plated at a density of 25,000 cells per well in a 96-well plate. After 24 h cells were transfected in triplicates using 10 µL of LNP-ASL mRNAs diluted to specific doses of 0.1, 0.5, and 1 µg/well using PBS in a total volume of 70 µL per well. Samples were collected on days 1, 4, and 6 by removal of supernatants and lysis of the cell layers in RIPA buffer containing protease inhibitors. Three wells were pooled per sample and centrifuged at 10,000 rpm for 12 min to remove cell debris. Samples were stored at −20 °C until further analysis.

Human buffy coats from healthy individuals were obtained from the Faculty of Medicine of Johannes Gutenberg University, Mainz. Peripheral blood mononuclear cells (PBMCs) were isolated by Ficoll-Paque™ PLUS (Cytiva, Marlborough, MA, USA) density gradient. Cryopreserved PBMCs were thawed and seeded into 96-well plates at a density of 5 × 10^5^ cells per well in 190 μL RPMI supplemented with 1% non-essential amino acids (NEAA), 1% sodium pyruvate and 10% fetal bovine serum (Merck, Darmstadt, Germany). After seeding, cells were transfected using a dose range of LNP-ASL mRNAs by adding 10 µL resulting in a total volume of 200 µL per well. After 24 h cells were centrifuged at 300 rpm for 8 min and supernatants were collected. Samples were stored at −80 °C prior to analysis.

### 2.3. In Vivo Delivery to Wild-Type and ASL^Neo/Neo^ Mouse Model of ASLD

All animal-related procedures were conducted at Genevant Sciences Corporation, an accredited facility, according to written operating procedures, in accordance with the Canadian Council on Animal Care (CCAC) Guidelines on Good Animal Practices and approved by the local Institutional Animal Care and Use Committee (IACUC). Rodent studies were performed under AUP numbers 0618001 and 0618002.

Male C57BL/6J (JAX stock 000664/) mice, 7–8 weeks old, were used for in vivo experiments deemed to be wild-type. Mice were acclimatized for 1 week upon arrival at the facility and randomly sorted into study groups. Any mice showing signs of injury or unusual behavior prior to the study were excluded.

To model ASLD in mice, we used the ASL^Neo/Neo^ mouse model in which a neomycin resistance cassette (Neo) inserted into the ASL gene disrupts its transcription [4]. Both male and female ASL^Neo/Neo^ mice were obtained in-house through breeding heterozygous unaffected mice (JAX stock #018830). This hypomorphic mouse model has less than 20% residual ASL activity, recapitulating the human ASA patients’ phenotype. The challenge with this mouse strain is that survival beyond three weeks is limited and only 25% of pups are affected by this autosomal recessive condition. Affected mice are phenotypically identifiable by reduced hair and size growth, and genotyping was used to confirm their status. Improving the survival of ASL^Neo/Neo^ mice was necessary to allow them to reach a sufficient size for intravenous dosing. To achieve this the weaning period was increased from 3 to 5 weeks and the diet was supplemented with a low-protein gel thereafter. To create a model that is clinically relevant, mice were switched to a high-protein diet at the beginning of the study. Body weights and long-term survival were greatly reduced due to the disrupted urea cycle and ammonia accumulation. Sex and body weight were considered when sorting mice into study groups with an equal number of each sex between groups and the starting group′s average body weights were kept within 10% of each other. Five mice per group were used for the high-protein challenge studies. Any mice showing signs of injury or unusual behavior prior to the study were excluded. Group treatments were not blinded to the staff.

LNP formulations encapsulating ASL mRNA were injected IV via the tail vein at the specified dose levels to mice (>8 weeks old). On the day of injection, the LNP stocks were filtered and diluted to the required dosing concentration with PBS, pH 7.4.

In wild-type mice at 6 h post-dose, blood samples were collected via a tail nick and processed to isolate plasma for cytokine analysis. At 24 h post-dose, animals were anesthetized with a lethal dose of ketamine/xylazine. Liver samples were collected, weighed, and flash-frozen in liquid nitrogen. Tissue samples were stored in FastPrep^®^ tubes at −80 °C until they were analyzed for ASL expression.

For the ASL^Neo/Neo^ mouse model of ASLD, animals were weaned at 5 weeks and supplemented with a low-protein diet gel thereafter. For the high-protein diet challenge study all food was replaced with a high-protein diet (Envigo 40% protein diet TD.90018). Animals were weighed daily to monitor their health status.

### 2.4. Western Blots, Jess, and Mass Spectroscopy Quantification

The amount of protein in whole-cell lysates was quantified using the Pierce BCA protein assay according to the manufacturer’s protocol. Western blots were performed using 3 µg of total protein per lane on 4 to 15% gradient Mini-PROTEAN^®^ TGX Precast Gels (Bio Rad, Hercules, CA, USA). ASL antibody ab201025 (Abcam) at a dilution of 1:1000 was used for the detection of ASL protein and anti-beta-actin HRP ab49900 (Abcam) diluted 1:50,000 or HSP90AB1 Mouse Monoclonal Antibody [Clone ID: OTI4C10] (OriGene) were used to detect the beta-actin and HSP90 loading controls, respectively. As a secondary antibody, DIANOVA 111-035-003 goat anti-IgG-HRP (BIOZOL) at a dilution of 1:5000 was used.

To obtain quantitative Western blot measurements, Jess system (Protein Simple), a capillary electrophoresis immunoassay, was used according to the manufacturer’s instructions. ASL protein was detected using an anti-ASL antibody, ab201025 (Abcam), in a dilution of 1:200; an anti-rabbit near-infrared (NIR) secondary antibody was used at a dilution of 1:20. Samples were detected in the NIR channel, and the amount of protein was normalized using the Protein Normalization Assay Module (Protein Simple).

ASL mass spectroscopy quantification was performed by Jade Bio (La Jolla, CA, USA) using a custom liquid chromatography–mass spectrometry (LC-MS)-based method. Briefly, the assay is performed on a targeted absolute protein quantitation platform. Using a triple–quadruple (QQQ) mass spectrometer, a tandem selection process whereby selecting peptides by precursor *m*/*z* is followed by selecting fragmentations (transitions) of the targeted peptide. Furthermore, coupled with liquid chromatography and the addition of heavy labeled internal standards the ASL protein is quantified.

### 2.5. Cytokine and Chemokine Assays

Meso Scale Discovery V-PLEX Custom Human Biomarkers Chemokine Panel (Meso Scale Diagnostics—MSD, Rockville, MD, USA) was used for the analysis of chemokine secretion in supernatants of human PBMC cultures according to the manufacturer’s instructions, with the sample dilution of 1:5. The levels of MIP-1β (macrophage inflammatory protein 1 beta), MCP-1 (monocyte chemoattractant protein-1) and IP-10 (interferon-gamma-induced protein 10 kDa) were quantified 24 h after the LNP-ASL mRNA transfection. In mice, neat plasma was analyzed 6 h after IV dosing using the Luminex™ 200™ instrument system. A custom ProcartaPlex™ panel was used according to the manufacturer’s instructions to quantify the cytokines.

### 2.6. Statistical Analysis

GraphPad Prism software 9.3 was used for the statistical analyses. Significant differences are indicated as * *p* < 0.05, ** *p* < 0.01, *** *p* < 0.001; ns equals no significance. One-way ANOVA was applied to compare three or more datasets along with the appropriate multiple-testing correction method. An adjusted *p*-value of <0.05 was considered statistically significant.

## 3. Results

### 3.1. mRNA Therapeutic for the Treatment of ASA

**Figure 1:**
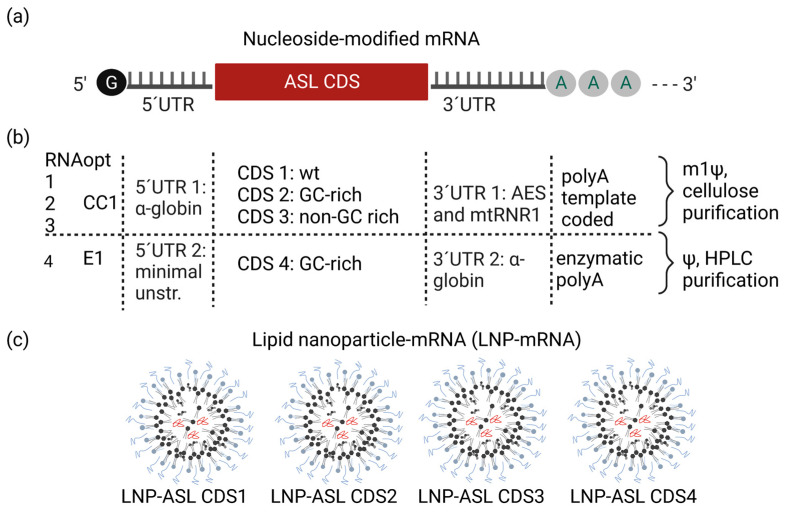
Development of mRNA-LNP therapeutic for treatment of ASA. (**a**) Schematic shows optimized nucleoside-modified mRNA composition: a cap structure, 5′ and 3′ untranslated regions, ASL coding sequence (CDS) as well as poly(A); (**b**) table compares the differences of mRNA constructs used for the experiments. CleanCap1 (CC1) was used for CDS 1, 2, and 3 or enzymatic cap1 (E1) for CDS4, 5′UTR 1/3′UTR 1 was used in CDS 1-3, 5′ UTR 2 (minimal unstructured UTR)/3′UTR 2 in CDS 4. CDS 1–3 include an encoded polyA tail and incorporated N1-methylpseudouridine (m1Ψ) instead of uridine (U), whereas CDS4 was enzymatically polyA tailed and includes incorporated pseudouridine (Ψ) instead of uridine (U). CDS1 represents the wild-type coding sequence (wt.) while CDS2-CDS4 are GC-rich or non-GC-rich optimized coding sequences; (**c**) each of the four modified mRNAs was formulated individually in the same type of lipid nanoparticle (LNP), forming an mRNA therapeutic for the treatment of ASA.

To design an optimal in vitro translated mRNA for the treatment of ASA we engineered diverse parts of mRNA to increase its efficiency and safety. First, to allow efficient translation after cellular uptake, each of the four in vitro translated mRNA optimizations was capped using a cap structure on its 5’end. We capped mRNAs using classical enzymatic capping (Cap1) or CleanCap1 co-transcriptional cap structure (Figure 1a,b).

The 5′ and 3′ untranslated regions (UTR) consist of numerous motifs, sequences, and structural elements that significantly regulate the translational efficiency of mRNA [25,34]. In our screening, we examined two different 5′UTRs and two 3′UTRs. The screening included alpha-globin 5′UTR and AES and mtRNR1 3′UTR that were previously used in the BioNTech-Pfizer COVID-19 vaccine and are known to contain motifs that increase the translation and stability of mRNA [26,27]. To ensure a full enhancement of translation, polyadenylation of mRNA at its 3′end is necessary [35]. To polyadenylate mRNA we used enzymatic polyadenylation or poly(A) template-coded poly(A) tail (as previously used in the BioNTech-Pfizer COVID-19 vaccine [25,27]). Second, to achieve improved safety of the therapeutic mRNA we used nucleoside-modified mRNAs, known to lower immunogenicity and minimize potential side effects caused by the release of proinflammatory cytokines and chemokines and we purified CDS1-CDS2 using cellulose purification and CD4 using HPLC purification [28,36]. To optimize the mRNA sequence encoding for the ASL enzyme we compared the wild-type sequence with three ASL sequences that were codon-optimized. Increasing guanosine: cytidine (G:C) content and replacing rare codons with more frequently occurring codons has been shown to enhance the translational efficiency of the mRNA. Thus, we designed two of the coding sequences (CDS) as GC-rich. The quality of manufactured ASL mRNA was high, with RNA integrity of 89, 92, 93, and 73% for CDS1 to CDS4, respectively (Table 1). Capping efficiency was measured as 84, 80, 87, and 99% for ASL mRNA CDS1 to CDS4, respectively, and all mRNAs passed the requirements having a minimal amount of double-stranded RNA (dsRNA) contaminants (Table 1) in line with quality control acceptance criteria for RNA-based therapeutics [37]. Finally, to be delivered to the target cells, each of the four optimized mRNAs was formulated using LNPs (Figure 1c). The LNP-mRNA had sizes optimal for liver targeting (72 to 77 nm), polydispersity index of 0.05 to 0.09, and mRNA encapsulation efficiency of 87 to 93% (Table 1) [37,38].

### 3.2. Robust Protein Expression of ASL mRNA Payloads In Vitro

**Figure 2:**
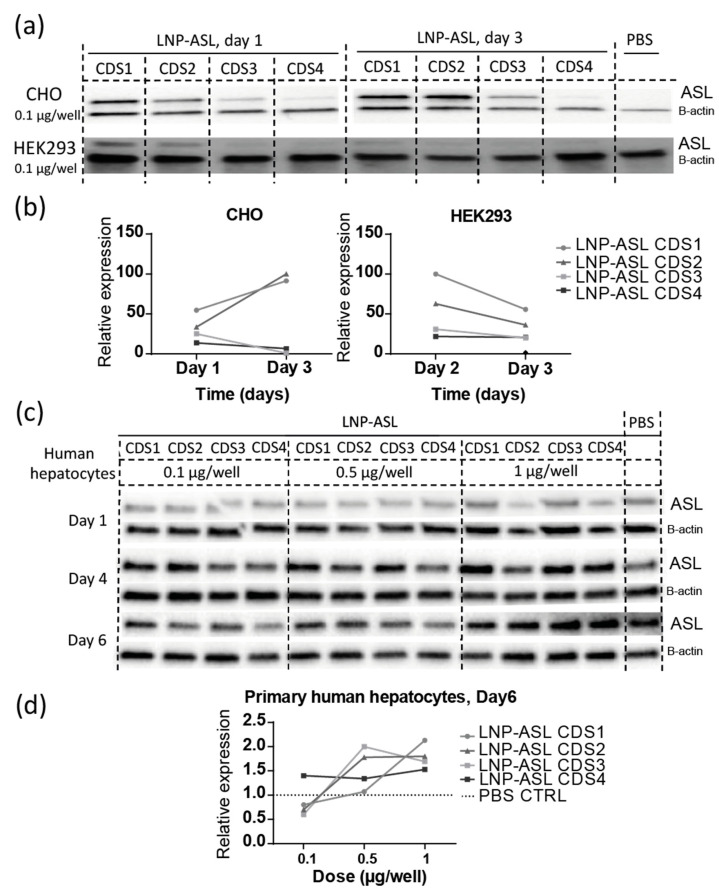
Robust protein expression of ASL mRNA payloads in vitro. (**a**) Western blot detection of ASL protein expression in CHO and HEK293 cells from Day 1 and Day 3 after transfection of 0.1 µg/well of four optimized LNP-ASL mRNAs. Beta-actin was used as a loading control and the same volume of PBS was applied instead of LNP-mRNA as a negative control; (**b**) relative expression of ASL protein to the PBS control was measured by Jess quantitative Western blot in CHO and HEK293 cells and showed a robust expression for all four optimized LNP-ASL mRNAs (CDS1–CDS4), with the strongest expression observed for LNP-ASL CDS1 (wild-type coding sequence) and CDS2 (GC-rich coding sequence) (individual samples, pools of four technical replicates); (**c**) Western blot analysis of four ASL-mRNAs (CDS1–CDS4) applied to primary human hepatocytes in a dose of 0.1; 0.5, or 1 µg/well from Days 1, 4, and 6. Beta-actin was used as a loading control and the same volume of PBS was applied instead of LNP-mRNA as a negative control, showing in this case amount of endogenous ASL; (**d**) relative expression of ASL protein to the PBS control was measured by Jess quantitative Western blot in primary human hepatocytes from Day 6 and showed a robust expression above control levels for all four optimized LNP-ASL mRNAs (CDS1–CDS4) when 0.5 or 1 µg/well was applied (individual samples, pools of three technical replicates). (**a**–**d**) Multiple conceptual replicates are shown.

To examine protein expression of LNP-ASL mRNA payloads in vitro we first applied four mRNA optimizations to CHO and HEK293 cells. Cell lysates were harvested on Day 1 and Day 3 after the transfection and Western blot analysis showed translation from all four ASL mRNA payloads (Figure 2a). No ASL was detected in phosphate-buffered saline (PBS) controls, as expected from these cell lines. To further quantify ASL expression we used Jess immunoassays in a capillary and further confirmed protein expression of all four candidates, with LNP-ASL CDS1 and LNP-ASL CDS2 showing substantially increased ASL protein expression (Figure 2b). We transfected the optimized LNP-ASL mRNA payloads to primary human hepatocytes and tested dose response at time points from one to 6 days (Figure 2c). In these cells, PBS controls showed endogenous human ASL levels. Western blot analysis using a standard approach showed expression of all tested LNP-ASL mRNA payloads (Figure 2c). Jess quantitative Western blot using a capillary approach resulted in the strong expression above the endogenous ASL background of all four LNP-ASL mRNA payloads when the applied dose was higher than 0.5 µg/well (7.1 µg/mL) (Figure 2d). At the dose level of 1 µg/well (14.28 µg/mL), the most robust expression detected six days after the transfection originated from LNP-ASL CDS1 and LNP-ASL CDS2, agreeing with the findings from other cell types examined (Figure 2d). Thus, in vitro studies revealed LNP-ASL CDS1 and LNP-ASL CDS2 as two lead-optimized mRNA candidates for the treatment of ASA. The most efficient translation in our in vitro model systems was achieved by optimizing RNA using human α-globin 5′UTR and AES/mtRNR1 3′UTR, with both wild-type (CDS1) and GC-rich (CDS2) coding sequence optimizations performing well. For example, mRNA compositions containing human α-globin 5′UTR and AES/mtRNR1 3′UTR with wild-type sequence and GC-rich coding sequence resulted in protein expression levels about 15-fold higher than those containing minimal unstructured 5′UTR and α-globin 3′UTR in CHO cells at day 3, and about 2-fold higher in HEK293 cells. In primary human hepatocytes, fold changes were up to 2.5-fold in all cases and were dependent on the dose. Optimizing the UTR regions strongly impacted protein expression, while coding sequence optimization from wild-type to GC-rich led to comparable high expression of both CDS1 and CDS2. However, comparing CDS1/CDS2 (wild-type and GC-rich) with CDS3 (non-GC-rich) showed that at day 3, CDS1/CDS2 expression was about 100-fold higher in CHO cells and about 2-fold higher in HEK293 cells, indicating that coding sequence optimization can also significantly impact the robustness of protein expression.

### 3.3. Low Cytokine Induction Post-mRNA-LNP Administration in Human Peripheral Blood Mononuclear Cells

**Figure 3:**
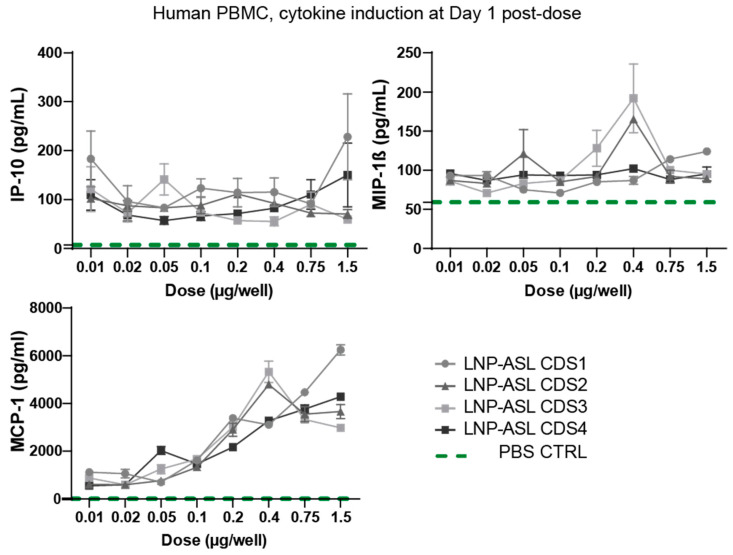
Low in vitro cytokine induction post-mRNA-LNP administration. Induction of interferon-gamma-induced protein 10 kDa, CXCL10 (IP-10), macrophage inflammatory protein-1 beta (MIP-1β), and monocyte chemoattractant protein-1 (MCP-1), 24 h after application of dose range from 0.01 up to 1.5 µg/well of four optimized LNP-ASL mRNAs was tested in human peripheral blood mononuclear cells (PBMCs) using Meso Scale Discovery. Control levels of cytokines in PBS-transfected cells are represented using a green dashed line. Legend refers to all three graphs in the figure. Data show mean ± standard deviation (SD) from two replicates (N = 2).

Safety is one of the key considerations in RNA protein replacement therapies, especially when repetitive injections are required over prolonged time periods or for life. The increasing amount of literature indicates that both LNP and mRNA components of mRNA-based therapeutics can induce immune activation [24,39,40]. The mRNA is sensed by numerous pattern-recognition receptors (PRRs) in the cell and LNP components are typically recognized by Toll-Like Receptor 2 (TLR2) and TLR4 located on the cell surface of macrophages and other cell types (reviewed in [24,41]). While this recognition triggers signaling cascades leading to cytokine and chemokine secretion and boosting the innate immune response evolved to have a role in anti-viral defense, it is unwanted when RNA-based protein replacement therapy of LNP-mRNA type is applied to patients since it may lead to temporary infusion reactions including fever and chills. Thus, we optimized our mRNA by sequence optimization and nucleoside modification to achieve minimal cytokine and chemokine secretion [42]. Additionally, a biodegradable and potent LNP developed at Genevant Sciences was selected for this therapeutic to minimize tolerability concerns around repeat dosing [43]. To test levels of immune activation after the application of the designed LNP-mRNAs for the treatment of ASA, we first transfected in vitro human peripheral blood mononuclear cells (PBMCs). We quantified cytokines/chemokines, as biomarkers of immunotoxicity, produced 24 h after the application of all four LNP-ASL mRNA in a dose range from 0.01 to 1.5 µg/well (0.5 to 7.5 µg/mL) (Figure 3).

Less than 250 pg/mL of interferon-gamma-induced protein 10 kDa, CXCL10 (IP-10), and macrophage inflammatory protein-1 beta (MIP-1β) was secreted after application of LNP-ASL mRNAs, independent of the applied dose. The lack of a dose response and nearly background levels of IP-10 and MIP-1β indicate minimal reactogenicity of human PBMCs to LNP-ASL mRNA-based therapeutics, predicting a high safety margin. As a positive control, we applied lipoplex-formulated unmodified RNA (LPX-uRNA) at a dose of 0.75 µg/well and found about 2-fold higher MCP-1 secretion, 30-to-46-fold higher IP-10 secretion and 82-to-106-fold higher MIP-1β compared to chemokine induction after application of LNP-ASL CDS1-CDS4 RNA (Figure S1), affirming that the human PBMCs used in this study were capable of pronounced immune responses. Monocyte chemoattractant protein-1 (MCP-1) secretion showed a dose dependence with the maximal secretion of 6.251 pg/mL when 1.5 µg/well of LNP-ASL CDS1 mRNA was applied. The chemokines tested in the human in vitro system were chosen as the most sensitive biomarkers of immunotoxicity known to play roles in cell-mediated immune responses and proinflammatory reactions [24,44]. The four LNP-ASL mRNAs (including mRNA harboring a wild-type coding sequence) were comparable regarding the chemokine response in human PBMCs.

### 3.4. Dose Response in Wild-Type Mice

**Figure 4:**
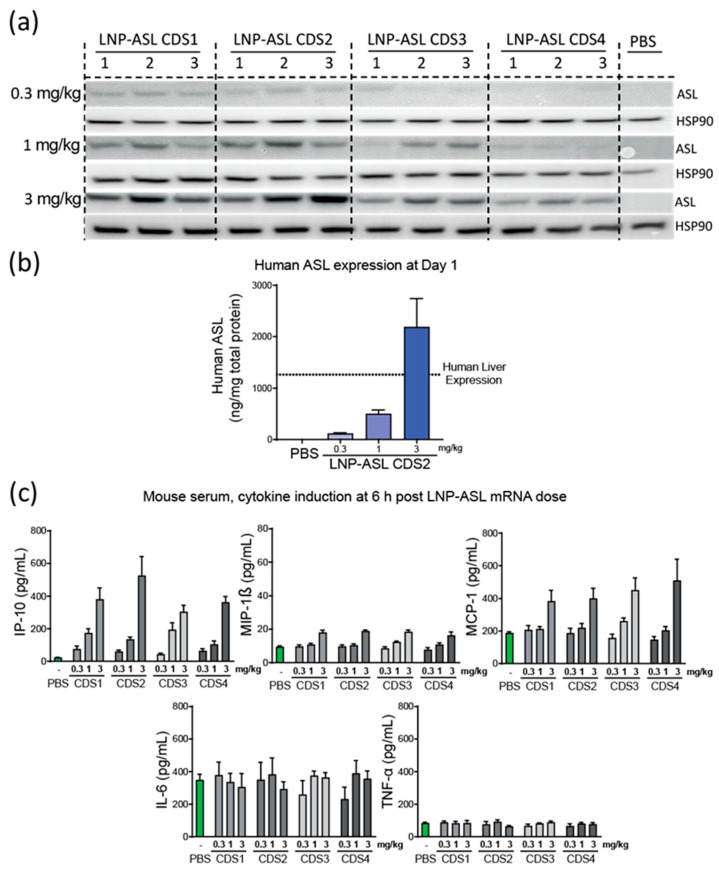
LNP-ASL CDS2 mRNA is highly expressed in wild-type mice and shows low cytokine/chemokine profile. (**a**) Western blot detection of ASL protein expression in mouse liver sample 24 h post-dosing (N = 3). Human heat shock protein 90 kDa alpha (HSP90) was used as a loading control and the same volume of PBS was applied instead of LNP-mRNA as a negative control; (**b**) the LNP-mRNA with the highest expression by Western blot was assessed by mass spectrometry for human ASL protein (N = 4). PBS-treated samples were analyzed as a control and endogenous human level of expression is plotted as a dotted line for reference; (**c**) Low cytokine induction post-mRNA-LNP administration in mice (N = 4/group). Induction of IP-10, MIP-1β, MCP-1, IL-6, and TNF-α 6 h post-dosing of 0.3, 1, or 3 mg/kg mRNA-LNP is shown. Control levels of cytokines in PBS-treated animals are represented by the green bars. Data show mean ± standard error of the mean (SEM).

To determine the lead mRNA payload in vivo, a dose–response study (0.3, 1, and 3 mg/kg) was conducted in wild-type C57BL/6 mice. Protein expression was evaluated in the liver of the mice 24 h post-LNP-mRNA dosing using a human-specific anti-ASL antibody. A dose-dependent increase in ASL expression was observed (Figure 4a). In agreement with the in vitro data, the highest expressing mRNA across all three dose levels were LNP-ASL CDS1 and LNP-ASL CDS2. Mass spectroscopy was used to confirm the expression profile of LNP-ASL CDS2 (Figure 4b). In agreement with the Western blot data, a dose-dependent increase in expression was clearly demonstrated, with the 3 mg/kg dose achieving expression levels above the endogenous liver expression in humans.

Cytokines were analyzed 6 h post-dosing to assess the safety of the mRNA-LNP (Figure 4c). No significant difference was observable between the mRNA payloads with both IL-6 and TNF-α showing no increase over PBS at any dose level. Minor increases (~2x) were observed at the 3 mg/kg dose level for both MCP-1 and MIP-1β. IP-10 showed a dose-dependent increase, but no notable difference between the mRNA payloads. The lead mRNA candidate was determined to be CDS2 when the totality of the in vitro and in vivo data was considered.

In addition to absolute expression levels, another important consideration for protein replacement therapeutics is the half-life of the protein. The longer the half-life, the less frequently the therapy would need to be administered. Previous data from Prieve et al. calculated that ornithine transcarbamylase, which is another urea cycle protein, has a half-life of ~12 days [29]. However, although these proteins are part of the same metabolic pathway, they could have different kinetics. To investigate the half-life of the ASL protein, 6 wild-type mice were administered LNP-ASL-CDS3 at 0.5 mg/kg. The livers from three mice were harvested 24 h post-dosing and the remaining three mice were sacrificed 7 days later. The expression at these two time points was quantified by mass spectrometry (Figure S2). Based on the mass spectrometry data, a half-life of ~5 days was estimated. This half-life estimate informed the selected dose frequencies that were investigated in the ASL disease model.

### 3.5. Repeat-Dose Efficacy of mRNA-LNP in ASLD Mouse Model

**Figure 5:**
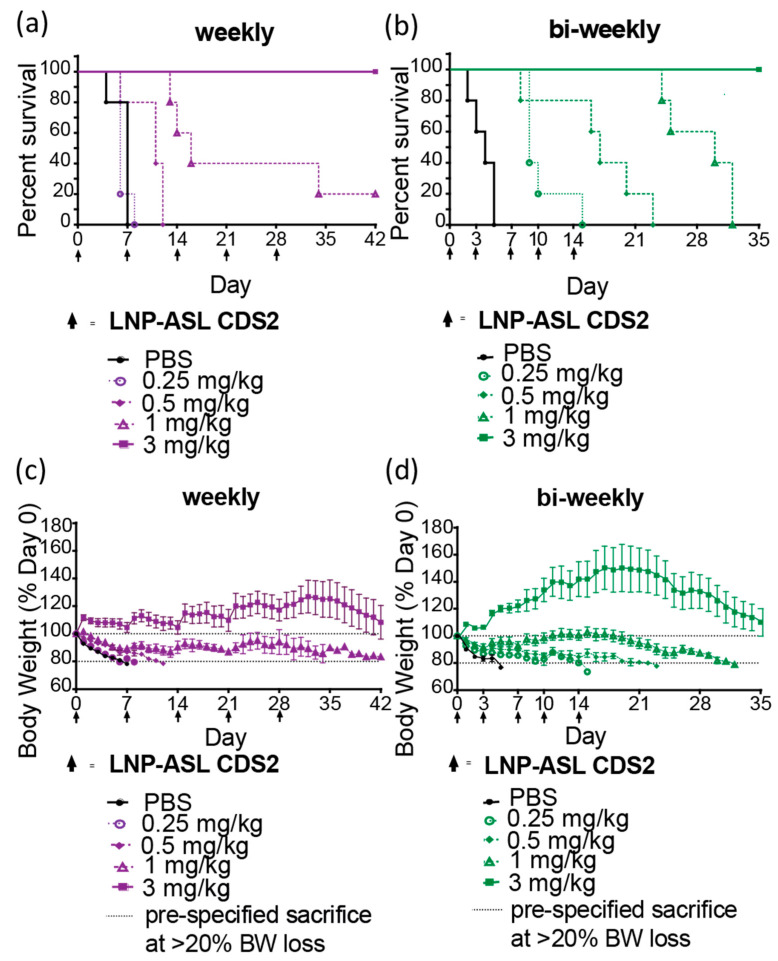
LNP-ASL CDS2 mRNA treatment leads to survival benefit in the ASLD mouse model. (**a**,**b**) Kaplan–Meier curves for ASL^Neo/Neo^ mice (N = 5/group) treated weekly or bi-weekly with LNP-ASL mRNA while challenged with a high-protein diet. Data from 4 dose levels and PBS control animals are shown; (**c**,**d**) the change in mouse body weight over time is shown for each dose group. A pre-specified loss of 20% body weight (BW) (dotted line) triggered animal sacrifice.

We next evaluated the efficacy of repeat-dose administration of LNP-ASL CDS2 in the ASLD model. ASL^Neo/Neo^ mice were switched to a high-protein diet [45] and treated with mRNA-LNP CDS2 or PBS on Day 0 (Figure 5a). The LNP-ASL CDS2 was administered at 4 different dose levels (0.25, 0.5, 1, and 3 mg/kg) in a once-a-week (OW) or twice-a-week (BIW) dosing regimen. The primary readout was daily body weight change, as impaired ureagenesis in combination with a high-protein diet would lead to rapid body weight loss in untreated animals. Dosing continued for 5 doses and any animal that met the pre-specified cut-off of 20% body weight loss was euthanized. The OW experiment was run for a total of 42 days and the BIW experiment was concluded after 35 days. In both experiments, the PBS-treated animals reached the experimental end point within 7 days. An obvious dose-dependent benefit to survival was evident with either dosing regimen (Figure 5a,b).

Animals treated with OW showed no protection at the 0.25 mg/kg dose level and partial protection at the 0.5 and 1 mg/kg dose levels. Full protection against the high-protein challenge was observed in the high-dose group (Figure 5a). This survival trend is supported by the changes in body weight over time (Figure 5c). It is evident that the top dose level was sufficient to maintain the body weight above the baseline throughout the OW dosing, although the body weights started to decline ~7 days after the last mRNA-LNP treatment. As expected, animals treated with BIW showed a better survival profile when under the high-protein diet challenge (Figure 5b). In this case, both the 0.25 and 0.5 mg/kg dose levels showed partial protection, while the 1 and 3 mg/kg showed full survival during the dosing (Figure 5b). Again, the changes in body weights agree with the survival data (Figure 5). There was a larger increase in bodyweight change for the 3 mg/kg dose level during the BIW dosing, with some animals gaining >50% of the initial starting weight. The lower dose levels also showed a slower decline in body weight loss compared to the OW treatments. The body weights began to decline ~7 days after the last mRNA-LNP treatment, underlining the importance of the expression kinetics of the delivered mRNA. As compared to weekly treatment, bi-weekly treatment of mice leads to increase survival and higher body weights. These effects were dose-dependent including plasma ammonium levels measured at 24 h after treatment (Figure S3). However, at later time points, a reduction of body weight was observed even for the highest-dosed group.

## 4. Discussion

ASA is a devastating rare genetic urea disorder usually evident and detected in the first few days of life, that would benefit from innovative treatment approaches. Patients are at an elevated risk of severe neurological impairment and even death when untreated [3,9,46]. Currently, the only available treatment strategies are based on dietary restrictions to limit protein intake, interventions to increase ammonia excretion, and liver transplantation [9,10]. However, most of the available therapeutics are not preventing but only managing symptoms and disease burden when toxicity already occurred, typically leading with time to multiorgan failures and progressive brain damage. Liver transplantation has been performed in affected patients; however, the procedure comes with a risk of mortality, risk of rejection and even if successful, still requires lifetime immunosuppression [15].

In this study, we show a demonstration of an mRNA-LNP therapy for the treatment of ASLD in a therapeutically relevant model using a non-viral delivery mechanism. Previous work has shown that AAV can also be used as a therapeutic approach in the murine ASL model but with limitations [6,16]. The benefit of an AAV-directed therapy is the duration of expression from a single treatment and the widespread expression in many cell types. However, there are several concerns regarding the use of AAV in ASLD patients. First, its durability in pediatric ASLD patients is limited as the growth of affected tissues can lead to a loss of efficacy over time [47]. Second, AAV vectors are susceptible to pre-existing neutralizing antibodies and can trigger cytotoxic T-cell responses to the viral vector that limit its effectiveness [47]. Furthermore, AAV is known to cause significant immune responses, which are treated with glucocorticoid drugs. Such drugs can trigger a hyperammonemic crisis upon catabolism by suppressing urea-cycle-related gene expressions and are therefore contraindicated in ASLD [48].

In this study, we incorporated N1-methylpseudouridine (m1Ψ) or pseudouridine (Ψ) into LNP-ASL CDS1-CDS3 or LNP-ASL CDS4, respectively. Pseudouridine (Ψ) increases the translational capacity and stability of mRNA, plus it significantly decreases immunogenicity compared to unmodified mRNA [42,49]. N1-methylpseudouridine (m1Ψ) may increase translation and lower immunogenicity even further as previously shown by Andries et al. [50]. We also found that GC-rich N1-methylpseudouridine-containing ASL mRNA translated better in comparison to GC-rich pseudouridine-containing ASL mRNA, however the nucleotide modification was not the only difference between these constructs and a positive impact from other mRNA features such as the UTRs cannot be excluded. The limitation of our in vitro experiments evaluating ASL mRNA is a lack of direct replicates. Individual samples were pooled from three to four technical replicates and then used for Western blots and Jess assays. However, regardless of this issue, all in vitro experiments we presented using diverse methodologies and various cell types indicated that LNP-ASL CDS1 and LNP-ASL CDS2 were the best two lead optimized mRNA candidates for the treatment of ASA, and this was independent of the in vitro model or method used. Furthermore, dose–response analysis of all four RNA optimizations in wild-type mice confirmed that the highest expressing mRNAs across the dose range were LNP-ASL CDS1 and LNP-ASL CDS2, providing confidence in the reproducibility of our findings. Importantly, we also demonstrated that delivery of an ASL-encoding optimized mRNA in an LNP formulation can protect a relevant in vivo disease model from the morbidity/mortality associated with ASA urea cycle disorder. mRNA-LNP shows good tolerability in vitro and in vivo. As demonstrated here all four mRNA candidates showed a favorable safety profile with relatively low increases in inflammatory cytokines at therapeutically relevant dose levels. The design of mRNA with incorporation of modified nucleosides as well as the inclusion of optimized sequences in the mRNA allows for maximal translation and low immune stimulation. The basic parameters such as mRNA quality and purity are universal factors for mRNA performance. The specific mRNA design and the suitable formulation are key factors, especially for tissue-specific delivery and translation, which should be tested out for each individual application according to needs and target cell type. To fully leverage the potential of ASL mRNA-LNP technology, further optimization may be necessary to refine the therapeutic window and enable full protection of animals at lower doses. It is worth noting that the presence of polyethylene glycol (PEG) in the LNP can trigger the formation of anti-PEG antibodies, which may reduce efficacy over time and lead to infusion reactions in some cases [24]. Therefore, exploring PEG-replacement options through future research could yield significant benefits.

In the field, there is some controversy over the complexity of ASLD and the impact of mutated ASL in the liver and in other organs and especially in the brain, on symptoms and severity of the disease. The basis for this controversy is in the role of ASL in two separate mechanisms: (1) the enzymatic role in the urea cycle in the liver which, when disturbed, typically leads to hyperammonemia and the associated sequelae; (2) the structural role in the citrulline–nitric oxide cycle in the brain [3,5,6]. The high levels of ammonia in the blood originating from urea cycle disruption in the liver is toxic to the central nervous system through the failure of potassium buffering in astrocytes, leading to increased extracellular potassium concentration and impaired cortical inhibitory networks [46]. As this impact of ASL mutation on the liver and brain through hyperammonemia is clear we applied our newly developed LNP-ASL mRNA to treat the liver-related toxicities. However, there is an unresolved controversy regarding which proportion of the neurological symptoms originatedfrom mutations of the ASL impairing its structural role in NO production in the brain and if treatment of the liver is likely to reverse the extrahepatic manifestation of the disease. Baruteau et al., 2018 for example showed in mice that cerebral disease in ASLD involves stress caused by NO cycle impairment independent of hyperammonemia and argued that simultaneous brain and liver gene transfer to treat both metabolic pathways at the same time is needed [6]. However, the number of human studies with liver transplants strongly suggests that treating the liver only already significantly alleviates not only liver-related but also neurological consequences. Ziogas et al., 2021 showed that 89.5% of ASLD patients that received liver transplants did not have encephalopathy after the transplant [15]. Newnham et al., 2007 also reported a case where prior to a liver transplant, encephalopathy was occurring about 5 times a year from the age of 15 leading to hospitalizations while full elimination of encephalopathy and no hospitalizations were observed despite unrestricted diet after the liver transplant at the age of 24 (follow up 30 months) [13]. In addition, improvement in diverse brain metabolism and neuropsychological assessments was observed indicating that neurological consequences of ASLD can be corrected at least in part by targeting the liver alone. Finally, in most of the ASLD cases with liver transplants strong improvement of quality-of-life was observed [13,15]. Thus, LNP-ASL modified mRNA therapy we developed for liver targeting is an important addition to the possible ASA therapeutics that may improve the survival and the life of patients.

## Figures and Tables

**Table 1:** Quality controls of ASL mRNA and lipid nanoparticle ASL mRNA (ASL mRNA-LNP).

Name (LNP-ASL mRNA)	mRNA Length (nt)	Codon Opt.	Bioanalyzer Integrity (%)	dsRNA per 1 µg mRNA (pg)	Capping %	Z-Avg (nm)	PDI	% Encap.
CDS1	1862	Wild-type	89	<1	84	73	0.06	88
CDS2	1862	GC-rich	92	<1	80	76	0.07	87
CDS3	1862	Non-GC-rich	93	<1	87	77	0.05	89
CDS4	1885	GC-rich	73	Pass	99	72	0.09	93

CDS, coding sequence; GC-rich, guanine, cytosine-rich; nt, nucleotide; codon opt., codon optimization; dsRNA, double-stranded RNA; Z-avg, Z-average size; PDI, polydispersity index; encap., particle encapsulation.

## Data Availability

The data presented in this study are available upon reasonable request from the corresponding author.

## Supplementary Materials

The following supporting information can be downloaded at: https://www.mdpi.com/article/10.3390/biomedicines11061735/s1, Figure S1: Human PBMCs are highly responsive to immunogenic stimulation; Figure S2: The estimation of the ASL protein half-life; Figure S3: Single-dose effect of mRNA-LNP on plasma ammonia levels in ASLD model.
