# Supplementary material for: ASL mRNA-LNP Therapeutic for the Treatment of Argininosuccinic Aciduria Enables Survival Benefit in a Mouse Model
Source: Biomedicines. 2023 Jun 16;11(6):1735. doi: 10.3390/biomedicines11061735 (PMC10296609; doi:10.3390/biomedicines11061735)
Supplement: Supplementary file 1 [file biomedicines-11-01735-s001.zip › biomedicines-2291789-supplementary.pdf]

Supplementary Figures

Figure S1

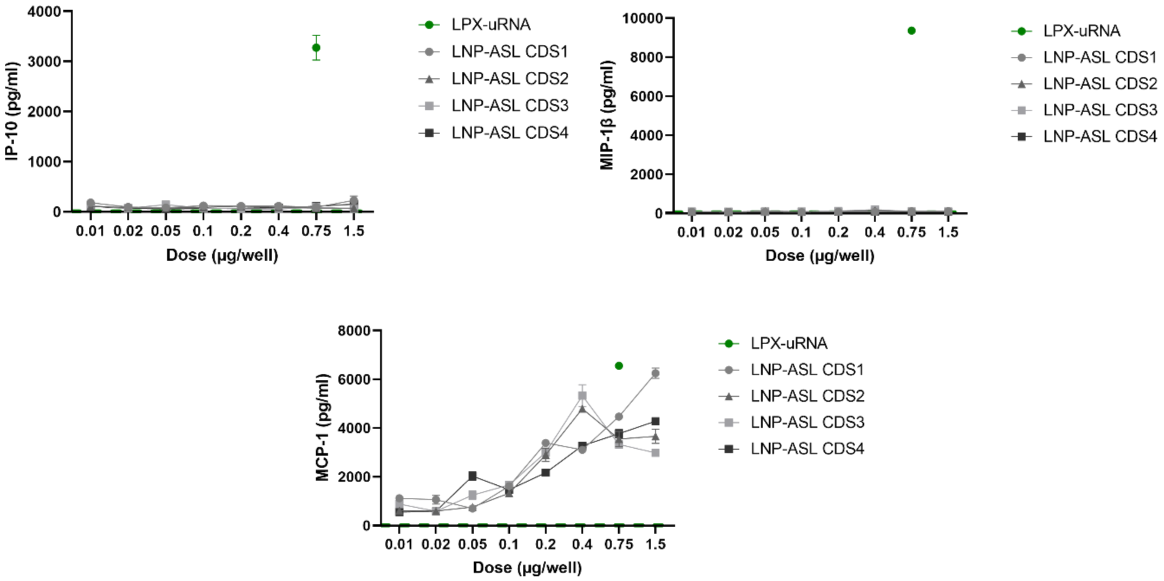

Human PBMCs are highly responsive to immunogenic stimulation. Induction of interferon-gamma induced protein 10 kDa, CXCL10 (IP-10), macrophage inflammatory protein-1 beta (MIP-1β), and monocyte chemoattractant protein-1 (MCP-1), 24 h after the application of a dose range from 0.01 to 1.5 µg/well of four optimized LNP-ASL mRNAs. The formulated mRNAs were tested in human peripheral blood mononuclear cells (PBMCs) and the cells responses evaluated using Meso Scale Discovery. As a positive control, 0.75 µg/well of lipoplex-unmodified RNA (LPX-uRNA) leads to a high cytokine secretion response in human PBMCs (green dot). Baseline levels of cytokines from PBS-treated cells are represented by a green dashed line. Data show mean ± standard deviation (SD) from two replicates.

Figure S2

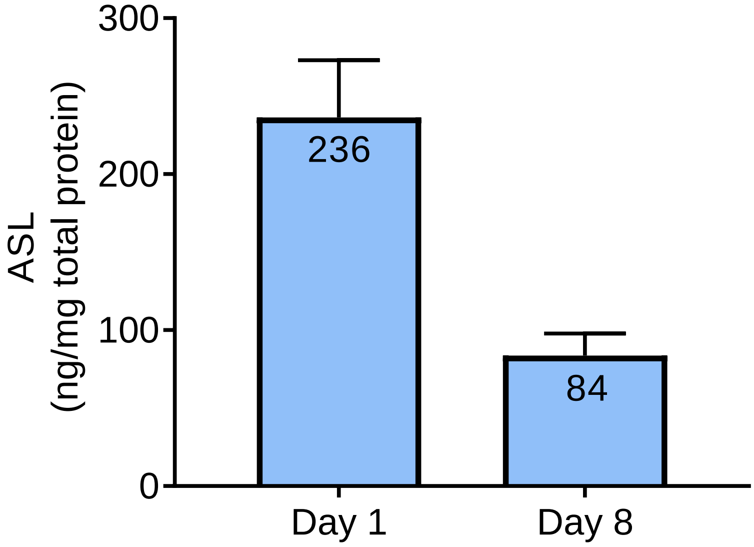

The estimation of the ASL protein half-life. Amount of ASL protein was measured using Mass Spectrometry in mouse liver (N=6) at day 1 and day 8 allowing estimation the ASL protein half-life of ~5 days.

Figure S3

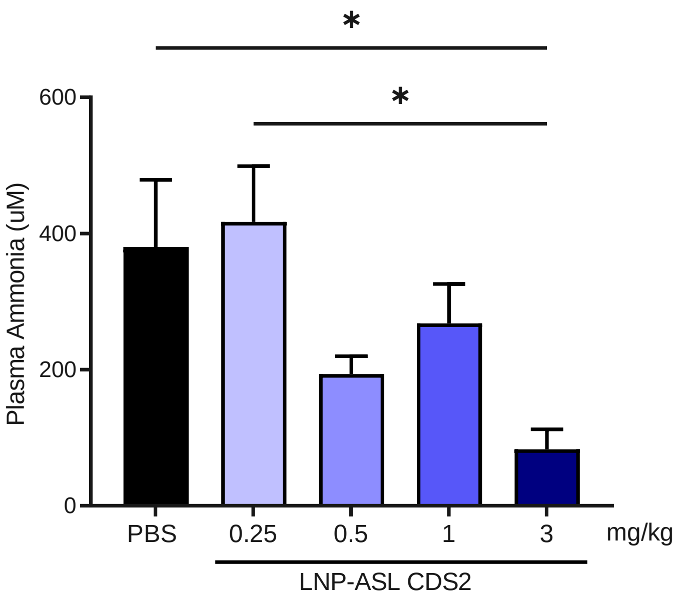

Single-dose effect of mRNA-LNP on plasma ammonia levels in ASLD model. The inability to clear nitrogen waste by urea synthesis in the liver leads to hyperammonemia in both patients and the mouse model of ASLD. To assess the effect of mRNA-LNP on plasma ammonia levels in $ASL^{Neo/Neo}$ mice, a single-dose study was performed at 4 dose levels (0.25, 0.5, 1, and 3 mg/kg). Mice were sacrificed 24 h post-dosing and the blood was collected to analyze plasma ammonia levels. A trend towards lower plasma ammonia with higher mRNA dose levels was evident although the difference to the PBS control only reached statistical significance at the highest dose level.

Methods

Plasma ammonia assay

Blood was collected by tail nick, processed to plasma, and stored at -80 °C until assayed. On the day of the assay the plasma was thawed on ice. Ammonia Assay Kit (Sigma, AA0100) was used to quantify ammonia levels. Plasma (10 µL) was added to 100 µL of Ammonia Assay Buffer and then the baseline absorbance at 340 nm was recorded. L-glutamate dehydrogenase (1 µL) was then added to each well, shaken gently and incubated at room temperature for 5 minutes. The absorbance at 340 nm was then recorded on a Tecan Safire 2 plate reader. Ammonia levels were calculated as described in the Sigma assay protocol.
